# Compressive Strength and Durability Properties of Structural Lightweight Concrete with Fine Expanded Glass and/or Clay Aggregates

**DOI:** 10.3390/ma11122434

**Published:** 2018-11-30

**Authors:** Deividas Rumsys, Edmundas Spudulis, Darius Bacinskas, Gintaris Kaklauskas

**Affiliations:** 1 Department of Reinforced Concrete Structures and Geotechnics, Vilnius Gediminas Technical University, Sauletekio av. 11, LT-10223 Vilnius, Lithuania; darius.bacinskas@vgtu.lt (D.B.); gintaris.kaklauskas@vgtu.lt (G.K.); 2 Institute of Building Materials, Vilnius Gediminas Technical University, Linkmenu str. 28, LT-08217 Vilnius, Lithuania; edmundas.spudulis@vgtu.lt

**Keywords:** expanded glass, expanded clay, lightweight aggregate concrete, microfiller, alkali–silica reaction, freeze–thaw resistance

## Abstract

This study is focused on the experimental investigation of compressive strength and durability properties of lightweight concrete mixtures with fine expanded glass and expanded clay aggregates using different microfillers. The paper proposes the relationships between the compressive strength and density of concrete mixtures with different proportions of the lightweight aggregates mentioned above. The performed experimental studies have revealed the tendencies of possible usage of different amounts of fine lightweight aggregates and their combinations in the production of concrete mixtures depending on the demands of practical application. Following the requirements for structural concrete subjected to environmental effects, durability properties (alkaline corrosion and freeze–thaw resistance) of the selected concrete mixtures with expanded glass aggregate were studied. The results of the experimental investigations have shown that durability of tested concrete specimens was sufficient. The study has concluded that the mixtures under consideration can be applied for the production of structural elements to which durability requirements are significant.

## 1. Introduction

Over the past few decades, the increased use of lightweight concrete for the structural elements of tall or long-span buildings has become a more and more important aspect in the modern construction industry. Lightweight aggregate concrete (LWAC) is not a new material. For many years, traditional aggregates (sand, gravel, etc.) have been replaced in concrete mixes with lightweight natural or human-made materials, or byproducts [1]. However, despite many advantages, practical application of such concrete in real structures is limited due to its lower mechanical properties and increased brittleness compared to normal weight concrete (NWC) [2]. On the other hand, a hardened mixture of the appropriate mechanical strength needs to be additionally tested to assess durability properties (freeze–thaw resistance, water absorption, etc.) in order to ensure the sufficient service life of structural members produced from this mixture [3].

The strength of the concrete mixture depends on the properties of the applied aggregate and cement mortar matrix. One of the ways to increase the strength of concrete is to use a fine aggregate in a concrete mixture by completely replacing the coarse aggregate. This is commonly used in production of high- and ultrahigh-strength concrete mixtures [4]. The application of the small diameter LWA (0.5–4 mm) in lightweight concrete allows the improvement of the homogeneity of concrete microstructures and the reduction of the possibility of segregation of the mixture [5]. Fine LWA is generally preferred to the coarse LWA due to a smaller distance between the aggregates. As a result, the internal curing water is provided to a greater volume of the mortar matrix [6]. Mechanical properties of the mortar matrix are affected by the water-to-cement (w/c) ratio, the amount of cement used, and mortar porosity [7,8,9]. The porosity of the matrix is usually reduced by adjusting the granulometric composition of the mixture and by introducing microfillers. The most commonly used and most effective microfiller is silica fume, which can increase the compressive strength of concrete up to more than 140 MPa [4,10]. Other microfillers, such as fly ash, limestone, siliceous microfiller, micronized phonolite, metakaolin, glass powder, etc., may also be used for mixtures [11,12].

The results achieved after many years of research in this field have shown that expanded clay is one of the most useful aggregates for the structural concrete [9]. Density and compressive strength of such concrete after 28 days of hardening is usually in the range of 1290–2044 kg/m^3^ and 23–60 MPa [9], respectively. Recently, as an alternative, expanded glass made from recycled glass waste [13,14] has been used for the production of concrete. Utilization of this type of aggregate in concrete as a main construction material is of primary importance for the sustainable development of the construction industry [15]. However, it should be emphasized that the application of the expanded glass aggregate (EGA) in concrete is only at its initial stage. Initial studies in this field have shown that the density and compressive strength of concrete after 28 days of hardening can be in the range of 1280–1490 kg/m^3^ and 23.3–30.2 MPa [13], respectively. The great advantage of expanded glass aggregate is the possibility to produce them in a much more varied fraction while maintaining a regular spherical shape. In the research reported by Yu et al. [13], up to five different fractions of EGA have been used in concrete mixtures. Four of them were up to 2 mm in size (a minimum fraction is 0.1–0.3 mm). Such a variety of particle sizes allows the selection of the optimal proportions of fine lightweight aggregate [16] and ensures the homogenous microstructure of hardened concrete.

The essential differences between expanded glass and expanded clay are due to their granulometry. In order to obtain a finer fraction of the expanded clay, it needs to be crushed. The use of crushed expanded clay aggregate (ECA) in the production of a concrete mixture is accompanied by higher water amount as well as a decrease in workability of the mix [9]. With increasing size of EGAs, their density decreases and is much lower in comparison to ECA of the same fraction [17]. However, too light aggregates complicate the production of the mixture and increase the probability of segregation [18].

From the point of view of durability of concrete, the alkali–silica reaction (ASR) represents one of the most important damaging mechanisms resulting in significant maintenance and reconstruction costs [19]. ASR occurs in concrete between alkalis from cement and siliceous constituents contained in the aggregate. The reaction leads to the formation of a calcium–alkali–silicate gel, which can lead to cracking, pop-outs, or expansion of a whole concrete element [20,21]. The test results of the rapid mortar bar expansion indicate that the replacement of 20% of Portland cement with ground glass powder (specific surface area—467 m^2^/kg) more than halves the expansion due to the alkali–silica reaction (ASR) [22]. Tuaum et al. observed that as the content of glass aggregate increased, ASR expansion also increased [23]. Similar processes can take place using a fine expanded glass LWA. Although EGA is of silicon origin, there is no detailed information on ASR of lightweight concrete with such aggregate. Some references indicate [24,25,26] that when using EGA in low-density concrete products, the ASR does not cause mechanical damage. The test results of the alkali–silica reactivity of the expanded aggregate based on glass, according to ASTM C 289, showed that the aggregate was highly reactive and was an additional source of alkalis [26]. However, it did not cause either expansion or cracks. Similar results were obtained using granulated foam glass. Specimens of a density of 750 ± 50 kg/m^3^ were produced and tested in order to determine ASR using a method similar to ASTM 1260 (1 M NaOH at 80 °C for 14 days) [25]. The resulting relative expansion amounted to 0.055% not exceeding limit values. The authors state that the ASR mechanism in concrete with LWA differs in that silica acid salts may accumulate inside the pores of aggregates, contributing only to its partial destruction inside porous aggregate particles [25]. The opposite results were obtained by testing ASR in specimens made using EGA of a diameter of 0–5 mm made by Penostek [27]. The RILEM TC 106-AAR method demonstrated that concrete with a density of about 500 kg/m^3^ did not meet the requirements for its relative expansion even using Aalborg white CEM I 52.5 R with Na_eq_—0.13%. Structural damage related to ASR is not characteristic of LWAC with the expanded clay aggregate. As can be seen, the ASR problem exists in products with EGA and Portland cement, and may occur differently in concrete of different composition or density.

Another aspect related to the durability of LWAC is the freeze–thaw resistance. The strength and water absorption properties of LWA can have a significant impact on the strength and freeze–thaw resistance of LWAC and is worse than that of normal-weight concrete [28]. Jones and Weiss have reported [29] that internally cured concrete with saturated lightweight fine aggregates shows a very low potential for freeze–thaw damage. It is characteristic of the cases when the amount of internally curing water is added to match the chemical shrinkage. The type of microfillers in concrete can also affect the freeze–thaw resistance. For concrete without a plasticizing agent, silica fume has significantly reduced the resistance of normal-strength concrete against the freeze–thaw effect. Meanwhile, fly ash showed better performance for concrete mixtures at a 0.40 W/C ratio [30].

In the present research, the investigation of lightweight concrete mixtures with fine expanded glass and expanded clay aggregates, as well as different microfillers such as silica fume and ground quartz sand, was carried out. The aim of the work was, first, to find out the relationships of the compressive strength and density using the low-fraction (0.5–1 mm) expanded glass aggregates and compare them with those obtained using expanded clay aggregates of larger fractions (2–4 mm) commonly used for structural lightweight concrete; then, to combine expanded glass and clay aggregates in various proportions while maintaining the uniform composition of the matrix mixture, and to observe the effect of proportions on compressive strength, density, and lightweight aggregate distribution in the mixture; and then to determine the influence of the expanded glass aggregate on the durability properties of the concrete mixture—alkaline corrosion and freeze–thaw resistance. The performed research is an additional step toward the development of lightweight concrete mixtures in order to increase their sustainable application in the construction industry.

## 2. Raw Materials and Test Methods

Main characteristics of materials used in this study are presented below:

**Cement:**
  - CEM I 52.5 R: density—3150 g/cm^3^; fineness (Blaine)—490 m^2^/kg; compressive strength after 28 days—61.1 MPa., K_2_O—1.07%, Na_2_O—0.10%.
**Fine aggregate:**
  - washed sand (S) fraction 0/4;
  - expanded glass LWA (EGA) fraction 0.5/1;
  - expanded clay LWA Leca-S (ECA) fraction 2/4.
**Microfillers:**
  - ground quartz sand (GQS); fineness (Blaine)—479 m^2^/kg; density—2605 kg/m^3^;
  - silica fume (MS), density—2216 kg/m^3^; chemical composition (% by mass): SiO_2_—96.06; Al_2_O_3_—0.20; Fe_2_O_3_—0.05; C—0.60; CaO—0.25; MgO—0.40; K_2_O—1.20; Na_2_O—0.10; SO_3_—0.35.
**Superplasticizer:**
  - superplasticizer based on polycarboxylate ester (No S1, No S3)—liquid;
  - superplasticizer based on polyethylene glycol (No S2)—powder.

**Figure 1:**
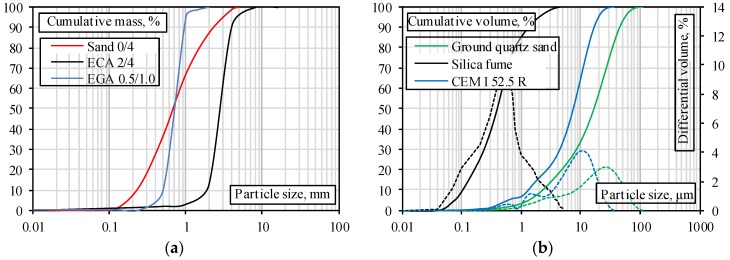
Granulometric curves of (**a**) aggregates; (**b**) microfillers and cement.

The characteristics were obtained using techniques of the following standards: bulk density of LWA was determined according to LST EN 1097–3 [31], water absorption after 24 h according to LST EN 13369:2013 (Annex G [32]), crushing resistance was determined by crushing according to LST EN 13055 (Annex C [33]), compressive strength of concrete specimens was determined according to LST EN 12390–3 [34], and the granulometry of a fine aggregate was determined by sieving method according to LST EN 933–1 [35]. The obtained data is presented in Figure 1a. The granulometry of the microfiller was investigated using the laser granulometer Particle Size Analyzer 1090 (Orleans, France). The main properties of the used aggregates are given in Table 1. The granulometry data of the microfillers are shown in Figure 1b.

All concrete mixtures were mixed using a 12 *l* capacity Hobart-type mixer (Brondby, Denmark). Mixtures were produced in the following sequence. At the initial stage, all the necessary materials were weighed. Then, all components and powdery superplasticizer were added to the mixing vessel. Afterward, water and liquid superplasticizer were gradually added, following with further mixing to obtain a homogeneous consistency. The prepared mixture was poured into standard shape steel forms (70 mm × 70 mm × 70 mm) without additional vibration. The specimens were then covered with a film and cured for one day. After a day, the specimens were demoulded, put into polyethylene bags, and cured for 28 days in a chamber at 20 ± 1.0 °C and 95% relative humidity. During the test, 120 samples were formed in total.

Prisms were taken out of the chamber after 28 days of casting, their dimensions were measured, and the weight was fixed. The compressive strength of the specimens was determined by the compression-testing machine ALHPA-3–3000 S using at least four specimens and having taken their average values. The loading speed used in tests was 2400 N/s. The density of the air-dried specimens was determined by dividing the resulting weight from the volume calculated according to the measured specimen sizes. The density was determined according to the average density of the measured individual specimens.

Alkaline corrosion of concrete samples was analysed using RILEM recommendations [36], while frost resistance tests were carried out according to the Lithuanian standard LST 1428–17:2016 [37]. Detailed information on the preparation of specimens for the latter studies and the research methodology is given in Section 4 and Section 5, respectively.

## 3. Effect of Fine LWA on Density and Compressive Strength of LWAC

The research program was divided into two different stages. The effect of fine EGA on the structural LWAC density and compressive strength was investigated during the first stage. The results obtained were compared to ECA mixtures. The composition of the mixtures under consideration as well as the compressive strength and density of concrete after 28 days of curing are presented in Table 2. In the present study, the reference concrete mix with 60% of sand by mass (mixes B1 and B2) was used. In mixes BG3 and BG4, 8.5% of the weight of sand was replaced by the same volume of EGA. The ratio between the volumes of sand and EGA was calculated according to the ratio of their bulk densities. BG5-BG12 compositions were made by changing 16.7, 33.3, 66.7, and 100 percent of sand mass, by analogy. By optimizing the granulometric composition of concrete, a different microfiller (silica fume or ground quartz sand) was used. The same program was repeated for BC3-BC12 mixtures by replacing EGA with an appropriate amount of ECA. In order to achieve sufficient workability and retain stable consistency of the mixture, a combination of two superplasticizers reducing the W/C ratio to 0.26 was used in all mixtures. Use of superplasticizer S2 has resulted in exceptionally good workability characteristics, however its excessive amount was causing accumulation of the free water on the surface of the mixture. Therefore, superplasticizer S1 was used to keep the uniform consistency and prevent separation of water from the mixture. The relationships between the compressive strength and density of concrete were proposed for each type of mixture.

**Figure 2:**
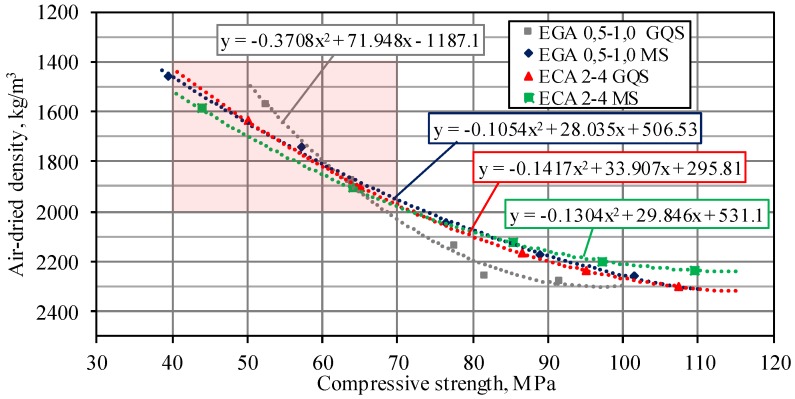
The compressive strength dependence on density for LWAC with EGA or ECA and different microfillers.

The obtained results have shown that in BG mixtures with EGA, the compressive strength after 28 days of curing varied from 39.5 to 101 MPa. Accordingly, depending on the amount of replaced sand, the density varied from 1458 to 2278 kg/m^3^. In mixtures with ECA, the compressive strength of LWA after 28 days varied from 43.8 to 109 MPa, and the density from 1588 to 2302 kg/m^3^. The compressive strength and density relationship of all specimens after 28 days of hardening is presented in Figure 2.

It can be seen from Figure 2 that very similar compressive strength and density relationships were obtained for different mixtures (the exception was EGA 0.5–1.0 GQS specimens). Although all the mixtures had the same amount of cement, W/C, and microfiller, as well as the same total volume of sand and LWA, a different compressive strength–density relationship was obtained for the EGA 0.5–1.0 GQS specimens having a combination of EGA and GQS microfiller. This is especially seen in low-density specimens with compressive strength greater than 50 MPa (BG11: compressive strength is 52.3 MPa and density is 1569 kg/m^3^). When a W/C ratio is low and the amount of LWA increases, the rheological properties of concrete have a tendency to change. As shown in Figure 1b, the silica fume microfiller consisted of particles of less than 1 μm and required more water to moisten them than a microfiller of ground quartz sand. Due to the high viscosity, the air entrained during mixing will have difficulty in escaping from the mixtures with MS microfiller. Viscosity of mixtures with GQS microfiller is smaller and, therefore, the microfiller is distributed more easily and uniformly. 

As can be seen from Figure 2, the compressive strength–density relationships developed in the study cover the cases of both ordinary and lightweight concrete. According to EN 206–1 [38], lightweight concrete includes concrete with a density of 800–2000 kg/m^3^ and compressive strength classes [39] between LC12/13 and LC80/88. The area of the Figure 2 representing lightweight concrete is noted in pink. Based on the data given in Table 2, it can be concluded that in order to obtain lightweight concrete with EGA 0.5–1.0 and ECA 2–4, at least 67% of the sand volume should be replaced by the same volume of LWA. If a smaller amount of sand is replaced by LWA, the reduction of concrete density is insignificant.

The unique research was carried out during the second stage. Expanded glass and expanded clay aggregates were mixed: the same total volume of LWA in a mixture was maintained and their interproportions were changed. The diameters of LWA used in the research differ approximately 4.0 times and the EGA 0.5/1 can fill the spaces between ECA 2/4. Their use together is a viable way to reduce the LWAC density and amount of cement. The composition of mixes with combined LWA is presented in Table 3. The composition of mixtures was calculated for concrete with a density of 1700 kg/m^3^. When comparing the influence of different LWAs and their possible combinations on the compressive strength and density in the reference mixture EGA0, lightweight aggregate was only expanded clay. In other mixtures (EGA5–EGA100) 5, 10, 20, 25, 50, 75, and 100% of the expanded clay were replaced with expanded glass. The numerical value of the specimen code provided in Table 3 indicates the EGA amount in a total volume of 100% LWA. The remaining LWA is ECA. Ground quartz sand and superplasticizer No S3 (2% of cement weight) were used as microfillers. Unlike in the first stage, saturation of 15 percent of the LWA mass was used to reduce the possibility of segregation. The saturated aggregate, due to the increased weight, has a lesser tendency to flow up to the surface of the mix. Moreover, the saturated aggregate is less able to absorb water from the mixture, thus safeguarding stable workability during placing. Saturation water was not included in W/C calculation. LWA was saturated in a closed container for 60 min before the production of the mixture. Saturation of LWA before the production of the mixture is also important in reducing the LWAC shrinkage during hardening due to the ability of porous LWA to absorb water while mixing and further moisture movement from the aggregate during hardening [40]. W/C for all mixtures was 0.33.

**Figure 3:**
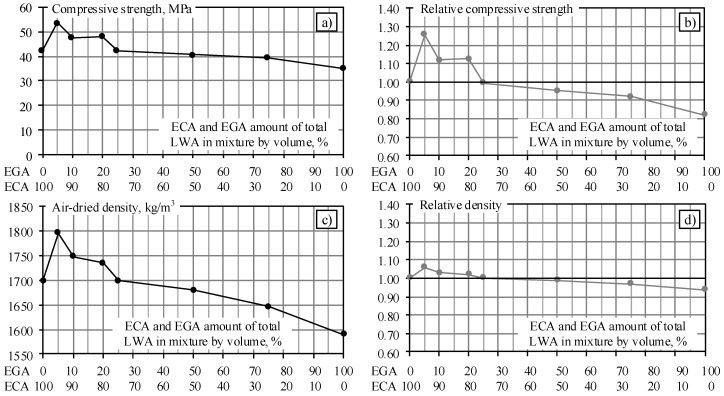
The compressive strength and density of LWAC with mixed LWAs after 28 days of hardening: (**a**) the dependence of compressive strength on the LWA ratio; (**b**) normalised compressive strength; (**c**) the dependence of air-dried density on the LWA ratio; (**d**) normalised density.

The relationship of the compressive strength and density for mixes with different amounts of ECA and EGA are presented in Figure 3a,c, respectively. For illustrative purposes, the relative compressive strength *f_cEGA0_/f_cEGA5...100_* and density ρ*_EGA0_/*ρ*_EGA5...100_* are shown in Figure 3b,d, where *f_cEGA0_* corresponds to the compressive strength of the concrete mix with ECA only and was taken as a reference value, whereas *f_cEGA5...100_* refers to the compressive strength of mixtures with an appropriate amount of EGA (from 5 to 100%); ρ*_EGA0_* is the density of the concrete mix with ECA only (reference density) and ρ*_EGA5...100_* is the density of mixtures with an appropriate amount of EGA.

The results above show that having replaced 5% of the ECA volume by EGA (sample EGA5), the density of LWAC increased from 1700 kg/m^3^ to 1796 kg/m^3^ (5.6%) and compressive strength from 42.7 MPa to 53.6 MPa (25.5%). The slight increase in density can be explained by the addition of a small amount of EGA (in diameter 4–8 times smaller than ECA). The spherical-shaped EGA particles behave like bearings that reduce friction between rough-surface ECA that, in turn, reduces viscosity of the mixture. Due to this, the entrained air during mixing escapes easier, which might assist in formation of a denser matrix. Addition of a small amount of EGA makes LWA packing tighter, which might also affect the internal curing process due to more evenly distributed water in the matrix. On the other hand, smaller particles of the aggregate could fill the pores contained in the mixtures, resulting in a higher-density mixture. However, it should be noted that a relatively slight increase in density is offset by a significantly higher compressive strength. Subsequently increasing the amount of EGA up to 25% in the mixture, the density of the reference mixture EGA0 and the EGA25 mixture becomes equal and the compressive strength varies very slightly (42.7 MPa and 42.4 MPa, respectively). A further increase of the amount of EGA in the mixture leads to a reduction in the strength and density of concrete. By increasing the EGA content in the mixture up to 50%, the compression strength decreases to 40.7 MPa (4.7%) and density decreases to 1680 kg/m^3^ (1.2%). In a mixture with 100% of EGA, the compressive strength decreases to 35.1 MPa (17.8%) and density to 1590 kg/m^3^ (6.5%). Since the total volume of LWA has not changed, the decrease in density occurs due to the decrease in the LWA weight with an increase of the amount of lower-density EGA in composition. Intermediate values of the compressive strength and density were obtained for the remaining mixtures.

Experimental studies of mixtures using different fine LWAs and their combinations have revealed the following trends. When concrete is used for a structural application that demands a higher compressive strength rather than a lower density, mixtures of EGA5, EGA10, and EGA20 may be used for the production of such concrete replacing ECA with EGA by 5, 10, and 20%, respectively. The compressive strength of these mixtures varies from 48 to 54 MPa, while the relative strength ranges from 1.12 to 1.26. When the compressive strength is not important, and the decisive factor is density (e.g., to produce a lighter structure), fine EGA is used as a lightweight aggregate in LWAC mixtures. The research results showed a density of 1590 kg/m^3^, which is 7% lower than the reference mixture of EGA0. In order to use mixtures with EGA for real structures, the durability properties, and in particular, alkali corrosion resistance, must be further evaluated. The research results of the durability of mixtures are presented in Chapters 4 and 5.

**Figure 4:**
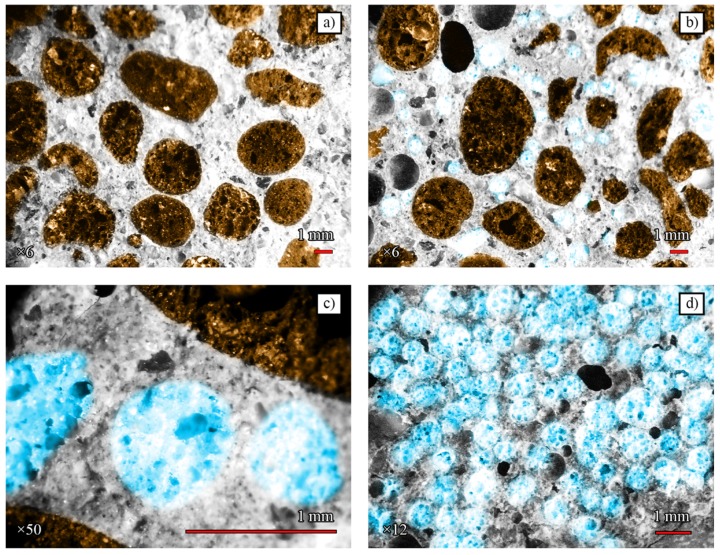
Microscopic photographs of specimens after splitting test (EGA highlighted in blue): (**a**) EGA0; (**b**) and (**c**) EGA5; (**d**) EGA100.

Placement in the matrix may vary when different LWA fractions are used. The structural analysis of samples using the MOTIC K-400 L digital microscope (Kowloon, Hong Kong) with a video camera Pixera VSC was performed additionally to evaluate this effect. Samples EGA0 (Figure 4a, magnification × 6), EGA5 (Figure 4b, magnification × 6 and Figure 4c, magnification × 50), and EGA100 (Figure 4d, magnification × 12) were scanned by a digital microscope in the course of research. Figure 4a shows that the dark ECA aggregates are surrounded by matrices from hydrated cement new growths and sand. However, relations between the surfaces of ECA particles are possible. The presence of EGA in the matrix (Figure 4b,c) reduces the number of interrelations between the ECA surfaces and the smaller-fraction EGA can affect the concrete mix in several ways: a) the reduction of friction between the larger ECA improves the flow of the mixture; b) water is distributed more uniformly in the concrete mix during the internal curing process. As the EGA quantity increases further (Figure 4d), there is no homogeneous matrix and aggregates form a connecting pair structure. This may lead to a decrease in the frost resistance of the mixture.

## 4. Alkali Corrosion Tests of Concretes with EGA

In addition to the mechanical tests, an experimental investigation on the durability (alkali corrosion and frost resistance) properties of the selected mixtures was carried out. The ASR intensity in concrete depends on many factors, but the key parameter is the amount of Na_2_O_eq_ in cement. It is calculated according to the formula: Na_2_O_eq_ = Na_2_O + 0.658 K_2_O [36]. Low-alkali cement has Na_2_O_eq_ = 0.10 + 0.658 × 1.07 = 0.804% (mass of cement). Two mixtures with ground quartz sand microfiller from the first stage of the study (Table 2) were used in the experimental investigation of alkali corrosion: a mixture without EGA (B1) and a mixture in which all sand had been replaced with EGA (BG11). These mixtures were chosen to determine the effect of different aggregates in ASR. Mixtures with silica fume microfiller have not been investigated because their particles are smaller than ground quartz sand and eliminate the effects of ASR [41]. The latter tendencies are also valid for ECA; therefore, mixtures with this aggregate were not analysed at this stage either.

The potential ASR in concrete was investigated using the accelerated mortar bar test and the RILEM AAR-2 method [36]. No additional amount of NaOH was added to the mixture. Therefore, RILEM requirements of 1.2% Na_2_O_eq_ [36] were not met (Na_2_O_eq_ = 0.804%). In the present study, the mortar prisms (40 mm × 40 mm × 160 mm) were cast using the technique presented in Chapter 2. After casting, the prisms were stored for 24 h at 20 °C temperature and 95% relative humidity. After demoulding, the prisms were stored for 24 h in water at 80 °C. Afterwards, their initial length was measured. Three prisms were then submerged in the 80 ± 2 °C 1M NaOH solution for 28 days. The recommended duration of ASR tests is 14 days [36]; due to the “short thick” prism forms, however, the duration of the test was increased to 28 days in order to obtain more reliable results. The effect of alkali corrosion is characterized by a relative elongation, the recommended limit value of which after 14 days of testing should not exceed 0.1% [36]. RILEM [36] additionally recommends a correction coefficient of 0.75–0.8 for the data obtained using “long thin” and “short thick” prisms. Therefore, taking into account the shape of the specimens, the threshold values of elongations decreased to 0.075–0.08%.

**Figure 5:**
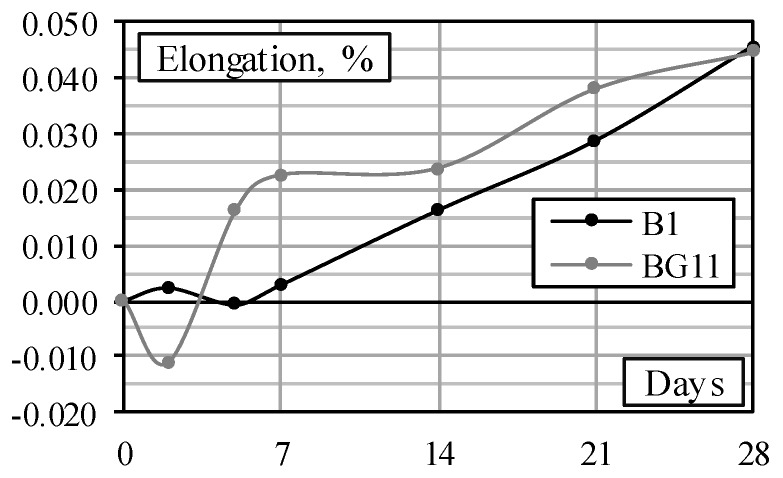
Expansion of specimens with sand (B1) and EGA (BG11).

The changes in the length of the prisms, determined experimentally, are shown in Figure 5. The figure shows that the contraction processes of both specimens occurring at the initial stage can be attributed to the cement hydration process [42]. Further processes can be associated with ASR. Due to the lower density, these processes occurred earlier in the BG11 specimen with EGA. After 28 days, the elongation of the specimens B1 and BG11 became the same.

Data on the change in the length of the prisms show that after 28 days of testing, the limit value of elongation obtained experimentally for both mixtures ~0.045% does not exceed the threshold value of 0.075% recommended by RILEM [36] (considering the effect of the form). It can be stated that using cement (Na_2_O_eq_ = 0.804%), LWAC specimens with fine EGA are affected by the ASR process to the same degree as the NWC specimens with sand aggregate. The studies carried out confirm that EGA in the LWAC mixtures can be applied without limitation related to ASR. Due to the lower EGA content, the same should apply to other LWAC mixtures analysed in this research.

## 5. Freeze–Thaw Tests of Concretes with EGA

Experimental investigations of frost resistance were performed with reference specimens without LWA, B1, and B2, and specimens where all sand was replaced by EGA, BG11, and BG12 (Table 2). Different microfillers were used in the tested specimens: ground quartz sand and silica fume.

For evaluation of the frost resistance of concrete in Europe, the technical specification CEN/TS 12390–9 [43] with three test methods can be used. One reference method is a slab test. Alternate methods are the cube test and the CF (Capillary Suction Frost) test. In this work, experimental tests of the frost resistance of concrete were carried out in accordance with the Lithuanian standard LST 1428–17:2016 [37]. This method is based on evaluation of changes in the compressive strength of the specimens after a certain number of freezing and thawing cycles. At least nine cube specimens must be produced in accordance with the requirements of the standard. Cube dimensions may be 150 mm, 100 mm, or 70 mm, depending on the size of the aggregate. Six specimens were used to evaluate frost resistance. The remaining cubes were used as reference for obtaining the compressive strength of concrete before carrying out cyclic frost resistance tests.

Nine concrete cube specimens of 70 mm were cast and further hardened for 1 day in the steel forms, and for 27 days in a polyethylene bag at a temperature of 20 ± 1 °C and 95% relative humidity. After 28 days of hardening, the specimens were immersed in water for 4 days. Afterwards, three reference specimens were removed and within 2 to 4 h were tested to obtain the initial compressive strength according to the LST EN 12390–3 standard [38]. The remaining specimens were frozen in air for at least 2.5 h at (−18 ± 2) °C and thawed in a water bath for at least (2 ± 0.5) h at (18 ± 5) °C. Three freeze–thaw cycles were performed per day, and 100 cycles were performed in total for each specimen.

The decrease in the compressive strength of concrete specimens after a freeze resistance test is calculated according to the following formula:

*Δf_c_* = (*f_k_* − *f_g_*)/*f_k_* × 100%, (1)

where *Δf_c_* is the decrease in the compression strength of concrete specimens compared to the control samples; *f_k_* is the mean value of the compressive strength of the control samples; *f_g_* is the mean value of the compressive strength of the specimens after a certain number of freeze–thaw cycles.

If the compressive strength of concrete specimens after the frost resistance test decreases by more than 5% compared to the reference compressive strength, the frost resistance of concrete is considered to be insufficient. The results of the frost resistance test carried out in accordance with the methodology described above are presented in Table 4.

All tested specimens withstood frost resistance tests up to 100 freeze–thaw cycles. The compressive strength of all samples, except BG11, after 100 freeze–thaw cycles was 2.0–7.4% higher than after 50 cycles. The increase in strength can be explained by the time factor. The compressive tests of control specimens were carried out before the frost resistance tests and others after a certain number of cycles. A further 20–40 days are required to complete the freeze–thaw cycle. If the freeze–thaw effect is not significant for the specimens, they continue to harden, and this results in the growth of their compressive strength. A decrease of the compressive strength of specimen BG 11 with GQS microfiller of 8.9% was observed after 100 cycles compared to the compressive strength after 50 cycles. This can be explained by the interaction of the microfiller and expanded glass aggregate. The GQS microfiller was larger than MS; therefore, the matrix structure obtained was more porous and weaker. This is also confirmed by the results of the BG11 and BG12 compressive strength tests carried out prior to the freeze test. During these tests, the compressive strength of the mixture with MS was 11.5% higher. As a result, due to the more porous matrix, water penetrated the specimen more easily through the formed capillary system and reached the lightweight aggregate. This significantly affected the frost resistance of concrete, and as a result, the loss of compressive strength occurred earlier. Using the MS microfiller (specimen BG12), the number of cycles increased the compressive strength evenly by 7.92%. The strength of control specimens with sand aggregate was 20.6% higher for mixtures with MS microfiller (B2) compared to mixtures with GQS (B1). This ratio slightly increased during freeze–thaw cycles. The strength of the B1 specimen has increased by 15.9% after 100 cycles of freezing compared to control specimens. Studies have shown that the durability of LWAC mixtures with expanded glass aggregate after 100 freeze–thaw cycles has been sufficient and can be applied for the production of structural elements (exterior walls or columns, bridge elements, etc.) to which these effects are relevant.

## 6. Conclusions

1. It was shown that in the case of the same composition of the cement matrix (a constant amount of cement, W/C, and microfiller) and the same total volume of aggregates (sand and LWA), the compressive strength and density relationships for different mixes are practically the same. However, in the specimens with EGA, the GQS microfiller slightly increases the effect of density on the compressive strength. The latter effect is caused by the difference in the particle size of GQS and MS, resulting in different rheological properties of the mixtures under a constant amount of water. In this case, GQS microfiller is more effective than silica fume microfiller.
2. Having replaced 5% of the ECA volume by EGA, the compressive strength has increased by 25.5% (from 42.7 MPa to 53.6 MPa) with a slight (up to 5.6%) growth in density of LWAC (from 1700 kg/m^3^ to 1796 kg/m^3^). This increase in density could be due to the reduced viscosity of the mixture caused by the small amount of EGA that, in turn, might affect the internal curing process due to more evenly distributed water in the matrix. On the other hand, smaller particles of the aggregate could fill the pores contained in the mixture resulting in a higher density.
3. An increase in the amount of EGA by 25% has practically no effect on the density and the compressive strength compared to the reference mix. A further increase in the amount of EGA leads to a reduction of the strength and density of concrete. By increasing the EGA content in the mixture up to 50%, the compression strength and density decrease by 4.7% and 1.2%, respectively. In a mixture with 100% of EGA, the compressive strength decreases by 17.8% and density by 6.5%. Since there is no change in the total volume of LWA, the decrease in density occurs due to the reduction in the LWA weight and the increase of the amount of lower-density EGA in the composition.
4. Specimens made of fine EGA and low-alkali cement (Na_2_O_eq_ = 0.804%) were affected to the same extent by the alkali corrosion test as the reference NWC specimens having the same cement. After 28 days of testing, the same elongation of 0.045% was obtained for both LWAC and NWC mixtures, the elongation being below the threshold value of 0.075% as recommended by RILEM.
5. It was shown that the durability of LWAC mixtures with EGA after 100 freeze–thaw cycles has been sufficient. The compressive strength of samples with EGA after the frost resistance test increased by 7.9–8.8% compared to the strength before the test.

## Figures and Tables

**Table 1:** Properties of aggregates.

Material	Bulk Density, kg/m^3^	Water Absorption after 24h, %	Crushing Strength, MPa
Expanded glass (0.5–1) mm diameter LWA	310	25.0	2.38
Expanded clay (2–4) mm diameter Leca S LWA	448	24.5	3.49
Sand (0–4) mm diameter	1624	–	–

**Table 2:** Mix proportions, compressive strength, and density of LWAC with EGA and ECA.

No.	Code	Quantity, kg/m^3^						(Super-Plasticizer/Cement) 100%	W/C	Compressive Strength, MPa	Density, kg/m^3^
		Cement	Sand	Silica Fume	Ground Quartz Sand	LWA					
						Type	Quantity				
1.	B 1	845	1410	-	95	-	-	No S1 3.0 + No S2 0.5	0.26	92.8	2347
2.	B 2		1410	95	-					102.5	2333
3.	BG 3		1290	-	95	EGA	23			91.4	2278
4.	BG 4		1290	95	-		23			101.4	2258
5.	BG 5		1175	-	95		45			81.5	2258
6.	BG 6		1175	95	-		45			88.9	2171
7.	BG 7		940	-	95		90			77.4	2138
8.	BG 8		940	95	-		90			76.3	2047
9.	BG 9		470	-	95		180			63.7	1877
10.	BG 10		470	95	-		180			57.2	1743
11.	BG 11		-	-	95		270			52.3	1569
12.	BG 12		-	95	-		270			39.5	1458
13.	BC 3		1290	-	95	ECA	33			107.4	2302
14.	BC 4		1290	95	-		33			109.4	2235
15.	BC 5		1175	-	95		65			95.0	2238
16.	BC 6		1175	95			65			97.0	2199
17.	BC 7		940	-	95		130			86.4	2167
18.	BC 8		940	95	-		130			85.3	2127
19.	BC 9		470	-	95		259			65.0	1901
20.	BC 10		470	95			259			64.0	1907
21.	BC 11		-	-	95		389			50.0	1637
22.	BC 12		-	95	-		389			43.8	1588

**Table 3:** Mix proportions of LWAC with mixed LWAs.

Code	Quantity, kg/m^3^			
	Cement	Sand	Ground Quartz Sand	LWA (ECA + EGA)
EGA0	608	525	152	360 + 0
EGA5				342 + 10.8
EGA10				324 + 21.6
EGA20				288 + 43
EGA25				270 + 54
EGA50				180 + 108
EGA75				90 + 162
EGA100				0 + 216

**Table 4:** Freeze–thaw test results of concretes with EGA and sand.

No.	Specimen	Compressive Strength, MPa			Changes in Compressive Strength *Δf_c_*, %	
		before Test	after 50 Cycles	after 100 Cycles	after 50 Cycles	after 100 Cycles
1	B1	108.0	115.8	125.1	+7.20	+15.90
2	B2	136.0	139.2	142.1	+2.30	+4.50
3	BG11	30.1	35.6	32.7	+18.30	+8.80
4	BG12	34.0	34.5	36.7	+1.30	+7.92
